# Cognitive reappraisal and expressive suppression strategies role in the emotion regulation: an overview on their modulatory effects and neural correlates

**DOI:** 10.3389/fnsys.2014.00175

**Published:** 2014-09-19

**Authors:** Debora Cutuli

**Affiliations:** ^1^Department of Psychology, University “Sapienza” of RomeRome, Italy; ^2^Laboratory of Experimental and Behavioral Neurophysiology, Santa Lucia FoundationRome, Italy

**Keywords:** emotion regulation, cognitive reappraisal, expressive suppression, brain volume, brain activation

## Abstract

Individuals regulate their emotions in a wide variety of ways. In the present review it has been addressed the issue of whether some forms of emotion regulation are healthier than others by focusing on two commonly used emotion regulation strategies: cognitive reappraisal (changing the way one thinks about potentially emotion-eliciting events) and expressive suppression (changing the way one behaviorally responds to emotion-eliciting events). In the first section, experimental findings showing that cognitive reappraisal has a healthier profile of short-term affective, cognitive, and social consequences than expressive suppression are briefly reported. In the second section, individual-difference findings are reviewed showing that using cognitive reappraisal to regulate emotions is associated with healthier patterns of affect, social functioning, and well-being than is using expressive suppression. Finally, brain structural basis and functional activation linked to the habitual usage of cognitive reappraisal and expressive suppression are discussed in detail.

## Introduction

The number of studies on emotion regulation has dramatically increased in the past two decades. These studies strengthened our knowledge on how the effectiveness of emotion regulation is crucial for different aspects of healthy affective and social adaptation (Gross, 2001; John and Gross, 2004). Further, dysregulation of emotions typically characterizes mood and anxiety disorders (Gross and Thompson, 2007).

Two major emotion regulation strategies that have been particularly studied are cognitive reappraisal and expressive suppression (Gross and John, 1998). In particular, *cognitive reappraisal* is defined as the attempt to reinterpret an emotion-eliciting situation in a way that alters its meaning and changes its emotional impact (Lazarus and Alfert, 1964; Gross and John, 2003). *Expressive suppression* is defined as the attempt to hide, inhibit or reduce ongoing emotion-expressive behavior (Gross and Levenson, 1993; Gross and John, 2003).

**Figure 1:**
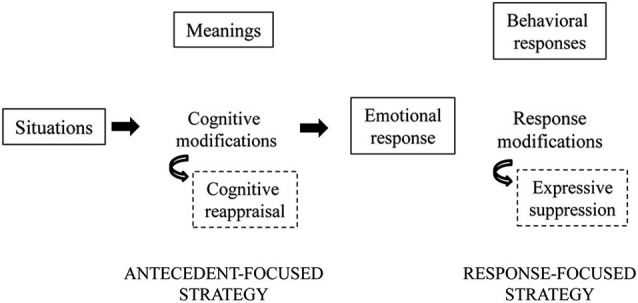
**A schematic representation of emotion regulation**. During the generative emotion processing, emotional situations can be managed modifying the emotional stimuli before the emotional response (antecedent-focused strategies) or still during the emotional response (response-focused strategies). In the first case cognitive modifications of the situation meaning can be used (i.e., cognitive reappraisal). Otherwise, a modulation of behavioral and physiological responses can be performed (i.e., expressive suppression). Here we are focusing on reappraisal and suppression strategies, anyway other antecedent-focused (e.g., situation selection and modification, attentional deployment) or response-focused (e.g., use of drugs, social sharing, relaxation) strategies can be used in regulating everyday affective experiences.

Based on an analysis of how emotions unfold over time, it has been argued that cognitive reappraisal and expressive suppression have their primary impact at different points of the emotion-generative process (Figure 1; Gross, 2001; Gross and John, 2003). Specifically, cognitive reappraisal is an antecedent-focused strategy that acts before the complete activation of emotion response tendencies has taken place. It thus might be expected to modify the entire temporal course of the emotional response before emotion responses have been completely generated. Expressive suppression is a response-focused strategy that intervenes once an emotion is already under way and after the behavioral responses have already been fully generated. It thus might be expected to require repeated efforts to manage emotional responses as they continually arise, challenging the individual’s resources.

The usage of cognitive reappraisal allows to implement and produce interpersonal behavior that is appropriately focused on social interaction and is perceived by the others as emotionally engaging and responsive. At odds, expressive suppression comes relatively late in the emotion-generative process and principally modifies the behavioral aspect of the emotional responses, without reducing the subjective and physiological experience of negative emotion, which is not directly targeted by suppression and may thus continue to linger and accumulate unresolved. As expressive suppression comes late in the emotion-generative process, it requires the individual to effortfully manage emotional responses as they constantly occur. These repeated efforts deplete cognitive resources to the detriment of social performances and create a sense of discrepancy between inner experience and outer expression in the individual (Higgins, 1987). The final effect of this sense of inauthenticity can lead to negative feelings about the self, making more difficult the establishment of emotionally close relationships and rather contributing to avoidant, diverted and anxious relational behaviors (Sheldon et al., 1997; John and Gross, 2004).

In the following sections, experimental findings on cognitive reappraisal and expressive suppression will be briefly analyzed. Then, individual-difference findings on the dispositional usage of these two strategies will be taken into account. Finally, brain structural basis and functional activation linked to the habitual usage of cognitive reappraisal and expressive suppression will be discussed in detail.

## Experimental studies

In experimental studies, participants are exposed to emotion-eliciting situations and randomly assigned to use cognitive reappraisal or expressive suppression strategies or to act naturally (control condition). Experimental studies use powerful research designs: in fact, by manipulating emotion-regulatory processes directly, they can demonstrate the immediate causal effects of particular strategies on dependent variables of interest, such as affective, cognitive and social consequences.

Overall experimental studies have demonstrated that cognitive reappraisal has a positive impact in the affective domain by decreasing negative emotion experience and negative emotion behavioral expression without any increase in physiological activation. At odds, suppression has a negative impact decreasing positive emotion experience and leaving unaltered the subjective negative emotion experience and exacerbating physiological activation (Gross and Levenson, 1993, 1997; Gross, 2002; Mauss et al., 2005; Hayes et al., 2010; Brans et al., 2013).

Cognitively, reappraisal results in unaltered or enhanced behavioral memory performance, while expressive suppression impairs memory performances (Richards and Gross, 1999, 2000; Dillon et al., 2007; Sheppes and Meiran, 2007, 2008; Hayes et al., 2010). Memory advantage for cognitive reappraisal may be subserved by the levels-of-processing effect (Dillon et al., 2007), which is characterized by deeper cognitive analysis of stimuli (Craik and Lockhart, 1972).

In experimental studies on the effects of emotion regulation strategies in social contexts, one member of each dyad is generally asked either to suppress, reappraise or to interact naturally with their conversation partner. When interacting with a person who was using suppression, subjects experienced more stress (i.e., greater increases in blood pressure) than when interacting with a person using reappraisal (Butler et al., 2003; Richards et al., 2003). Thus, while reappraisal has not detrimental effects, the cognitive costs of expressive suppression may concurr to compromise social functioning, as the suppressor fails to take up information needed to respond appropriately to the others and appears not tuned with the flow of the interaction.

## Individual difference studies

Since experimental studies cannot account for the long-term, cumulative consequences of using particular regulatory strategies for the individual’s emotional life, relationships and wellbeing, a complementary, correlational research approach was used. To this aim Gross and John (2003) developed a self-report questionnaire, the Emotion Regulation Questionnaire (ERQ), to assess individual differences in the usage of habitual, dispositional cognitive reappraisal and expressive suppression. Studies using ERQ have shown that the habitual use of these strategies varies systematically between individuals and is stable in time (Gross and John, 2003). Furthermore, cognitive reappraisal and expressive suppression resulted scarcely related to intelligence, social desirability and personality traits, but highly related to the constructs of inauthenticity, coping with stress and mood management (John and Gross, 2004).

Affectively, the use of cognitive reappraisal in everyday life is related to greater experience and expression of positive emotions and lesser experience and expression of negative emotions. By contrast, individuals frequently using expressive suppression experience and express less positive emotions, without differences in the negative ones (Gross and John, 2003; Abler et al., 2010; Larsen et al., 2012). However, expressive suppression may increase negative affect through its strict link with inauthenticity, specifically leading to feel bad about the self and even to depressive symptoms (John and Gross, 2004).

Cognitively, reappraisal has not effects on mnesic performances, while suppression is negatively related to memory, in particular for socially relevant information (Richards and Gross, 2000; Egloff et al., 2006; Hayes et al., 2010; Moore and Zoellner, 2012). In the domains of interpersonal functioning and well-being, cognitive reappraisal was interestingly associated with better psychological health. In fact, individuals who habitually use reappraisal showed lower symptoms of depression, were more satisfied and optimistic, and had higher self-esteem, environmental mastery levels, personal growth, self-acceptance, coping skills, sense of autonomy as well as better interpersonal relationships (Garnefski et al., 2001; John and Gross, 2004). At odds, suppressors feel to have less social support, worse coping abilities, lower life satisfaction, self-esteem, optimistic attitude about the future, higher avoidance and lack of close social relationships and support, all factors increasing the risk for depressive symptoms (Sheldon et al., 1997; John and Gross, 2004). Anyway, interesting recent studies demonstrated that culture has to be a moderator variable of emotion regulation, being the relation between expressive suppression and negative indicators of mental health stronger in the Western culture than in the Eastern one (Soto et al., 2011; Hu et al., 2014).

## Neural correlates of cognitive reappraisal and expressive suppression

As the habitual use of emotion regulation strategies shows stable individual differences, it could be possible that these strategies, either as a *consequence* (i.e., pre-existing individual volume differences lead to differences in emotion regulation) or *precondition* (i.e., brain region volumes are affected by the usage of emotion regulation strategies) are associated with individual differences in brain volumes and functional activation. Several studies have investigated the underlying neurobiological substrates of cognitive reappraisal and expressive suppression usage.

Following an overview of the studies on brain structural and functional variations associated to the use of cognitive reappraisal and expressive suppression is presented.

### Brain structural studies

In a magnetic resonance imaging (MRI) study, Welborn et al. (2009) investigated the relation between sex differences in orbitofrontal cortex (OFC) subregions and affective individual differences in healthy adults. As previously reported (Gross and John, 2003), women reported using suppression less frequently than did men. Volume differences based on participants’ gender were also identified with men showing larger left planum temporal and women showing larger ventromedial prefrontal cortex (vmPFC), right lateral OFC, cerebellum and basal ganglia. Strikingly, vmPFC (but not OFC) volume was positively related to individual differences in cognitive reappraisal and negatively related to expressive suppression usage. Further, vmPFC volume fully mediated sex differences in emotion suppression and partly in cognitive reappraisal.

In another region of interest (ROI)-based neuroimaging study, Giuliani et al. (2011a) found a positive correlation between cognitive reappraisal and the volume of the dorsal anterior cingulate cortex (dACC), but not the ventral ACC, in healthy female subjects. No relations between dACC volume and expressive suppression, negative affect or age were found. Given that expressive suppression is an emotion regulation strategy that requires interoceptive and emotional awareness, the role of anterior insula in this process was further investigated (Giuliani et al., 2011b). It was demonstrated that anterior insula volume positively correlates to expressive suppression, but not with cognitive reappraisal and negative affect. These findings are consistent with the idea that trait patterns of emotion processing are related to brain structure and indicate that individual differences in cognitive reappraisal are related to different dACC volumes, while individual differences in expressive suppression are related to different anterior insula volumes.

Using an exploratory whole brain approach, Kühn et al. (2011) examined the structural correlates of the habitual use of expressive suppression of emotions. They found a positive correlation of right dorsomedial prefrontal cortex (dmPFC) volume with expressive suppression, but no association of any other brain area with cognitive reappraisal. As expected on the basis of the important role that dmPFC plays in self-control and voluntary inhibition of action (Brass and Haggard, 2007; Brody et al., 2007; Campbell-Meiklejohn et al., 2008; Kühn et al., 2009), the response-focused emotion regulation strategy of expressive suppression is associated with increased gray matter volume in the dmPFC. Even if it is not possible to rule out that the increased dmPFC volume in subjects with expressive suppression strategies is an *a priori* condition rather than a consequence of behavior, it could be speculated that expressive suppression is under internal control as consequence of the internalization of societal norms, customs and manners that govern the adequate or undesirable emotional expressions.

Recently, using a voxel-based morphometry (VBM) in a large sample of young individuals it was analyzed the association of gray matter volumes of the a priori ROIs, including amygdala, insula, dACC/paracingulate cortex, medial and lateral PFC, with cognitive reappraisal and expressive suppression usage as well as neuroticism (Hermann et al., 2013a). Interestingly, a positive association of cognitive reappraisal and neuroticism with amygdala volume was observed. Furthermore, expressive suppression resulted positively associated with dACC/paracingulate cortex and medial PFC gray matter volume. These findings underline the role of the amygdala in individual differences in cognitive reappraisal usage as well as neuroticism that was not found in previous studies. Additionally, the association of expressive suppression usage with larger volumes of the dACC/paracingulate cortex and medial PFC underpins the role of these regions in regulating emotion-expressive behavior. It is evident that Hermann et al. (2013a) did not replicate previous results regarding greater dACC (Giuliani et al., 2011a) and vmPFC (Welborn et al., 2009) volume in frequent using cognitive reappraisers, and larger insula (Giuliani et al., 2011b) and smaller vmPFC (Welborn et al., 2009) volume in individuals frequently using expressive suppression. In contrast, the positive correlation of expressive suppression with dACC/paracingulate cortex and with vmPFC gray matter volume is in line with the involvement of dmPFC in the network linked to the inhibition of actions (Kühn et al., 2009).

Although somewhat conflicting, overall brain structural studies demonstrate that distinct brain structural variations of gray matter volume in the amygdala, insula, dACC, vmPFC and dmPFC might underlie individual differences in cognitive reappraisal and expressive suppression usage. However, a replication of these results is still missing because most of the abovementioned studies focused on different brain regions. Additionally, methodological factors (e.g., VBM *vs.* ROI approach) as well as sample characteristics (e.g., gender and age of participants) prevent a reasonable comparison of the results.

### Brain functional studies

The neural basis of emotion regulation processes have been further investigated by several functional neuroimaging studies by manipulating emotion regulation strategies (Ochsner and Gross, 2005). Generally, negative affective pictures are used and participants are trained to reduce the emotional impact of the pictures by using cognitive reappraisal. It is well known that not all individuals experiencing adverse experiences develop anxiety disorders, as result of individual differences in the regulation of negative emotions. Anyway, a more frequent use of habitual (dispositional) cognitive reappraisal in daily life has been shown to be more adaptive. Interestingly, the down-regulation of negative emotions through cognitive reappraisal is indicated by increased activation of medial and lateral PFC along with a diminished activation of emotional arousal-related brain structures as amygdala and insula (Ochsner and Gross, 2005; Ochsner et al., 2012).

Furthermore, dispositional reappraisal has been associated with reduced insula, hippocampus and amygdala as well with stronger dACC and dorsolateral PFC activation in response to aversive emotional stimuli (i.e., pictures or faces; Drabant et al., 2009; Carlson and Mujica-Parodi, 2010; Hayes et al., 2010; Vanderhasselt et al., 2013; Hermann et al., 2014).

Recently, the correlation of habitual cognitive reappraisal usage with stronger down-regulation of amygdala activation during instructed emotion regulation was reported also in a group of patients with remitted depression and healthy controls by using functional MRI (fMRI; Kanske et al., 2012). Hermann et al. (2013b) found that dental phobic individuals with higher dispositional cognitive reappraisal scores showed a reduced activation of the right dmPFC and increased activation of the right vmPFC and the lateral OFC over the course of symptom provocation. Cognitive reappraisal was a predictor of habituation during exposure to phobic stimuli rather than symptom severity. Given that extinction learning as well as cognitive reappraisal are crucial components of exposure-based cognitive-behavioral therapy (CBT) of phobias, the findings by Hermann et al. (2013b) point out for the special importance of considering individual differences in general cognitive reappraisal abilities of phobic patients prior to exposure sessions and to improve these abilities if necessary in order to strengthen the (long-term) outcome of CBT.

Up-to-date few studies examined the neural correlates of expressive suppression in response to emotional stimuli (Ohira et al., 2006; Goldin et al., 2008; Hayes et al., 2010; Vanderhasselt et al., 2013). Ohira et al. (2006) demonstrated a reduced amygdala activation during suppression of emotions. In a further PET study, Goldin et al. (2008) demonstrated increased PFC, insula and amygdala activation during the suppression of disgust facial reactions in response to disgust-eliciting film clips. Individual differences in expressive suppression usage have been further associated with higher amygdala activation when inhibiting responses to sad *vs.* happy facial expressions (Vanderhasselt et al., 2013). Suppressing facial expressions in response to negative picture engaged bilateral insular cortex, supramarginal gyrus and middle frontal gyrus (Hayes et al., 2010).

In parallel with gray matter volume studies, taken together these studies on the functional activation during cognitive reappraisal and expressive suppression confirm that differential activation of the amygdala, insula, dACC, PFC and OFC might underlie individual differences in the use of different emotional strategies.

## Discussion

Altogether experimental and individual difference studies underpin the crucial role of cognitive reappraisal and expressive suppression in adaptive as well as dysfunctional emotional processing and regulation. Furthermore, brain structural and functional studies depict a resulting brain network constituted by target regions for several emotional regulation processes. Namely, the amygdala has a crucial role in emotion regulation as it processes sensory information from the thalamus and somatosensory cortex and has bidirectional projections with hippocampus (emotional memories) and hypothalamus (physiological activation). The regulation of emotional processes is modulated by an rich net of interconnections among amygdala, insula (enteroception, sense of self) and the cortico-subcortical circuits of the OFC (saliency evaluation of emotional state, selection of adequate behaviors) and ACC (emotional state interpretation, motivated behavior). Also PFC (executive functions, cognitive elaboration) indirectly participates in the emotional regulation through its connections with OFC.

Not by chance association between amygdala gray matter volume and anxiety-related traits/states have been reported in numerous studies in healthy subjects (Barrós-Loscertales et al., 2006; Tottenham et al., 2010; van der Plas et al., 2010; Gerritsen et al., 2012) as well as altered activation and volume in the amygdala are common findings in mood and anxiety disorders (Etkin and Wager, 2007; Drevets et al., 2008; Irle et al., 2010; Atmaca, 2011; Kempton et al., 2011; Sacher et al., 2012). Furthermore, reduced activation of the vmPFC along with amygdala hyperactivation and a dysfunctional recruitment of ACC and dmPFC has been observed in patients with specific phobia and post-traumatic stress disorder (Schienle et al., 2007; Hermann et al., 2009; Milad et al., 2009), most likely indicating reduced cognitive control of emotional reactions. Interestingly, phobic individuals more frequently using cognitive reappraisal have an increased vmPFC activation during extinction learning and recall (Hermann et al., 2013b), probably related to a stronger extinction learning as following a successful CBT (Schienle et al., 2007).

The top-down emotional control network via cognitive reappraisal engages also OFC (Ochsner and Gross, 2005; Hermann et al., 2013b). By contrast, habitual bottom-up use of expressive suppression rely more heavily on the anterior insula (Giuliani et al., 2011a) and dACC/paracingulate cortex and medial PFC volume (Hermann et al., 2013a) as well as on increased insula, PFC and amygdala activation (Ohira et al., 2006; Goldin et al., 2008; Hayes et al., 2010; Vanderhasselt et al., 2013). In this neural correlates pattern the role of the insula emerges, not only in primarily supporting interoception and monitoring emotional awareness and outward emotional expression, but also as a relay point between the bottom-up signals from brain regions involved in emotional responding and inward emotional state, like the amygdala, and bottom-up signals from other regions involved in cognitive regulation and regulation goals, like the PFC (Nunn et al., 2008).

## Conclusions

As conclusive considerations, further studies are required to outline more in depth the relations among structural and functional data, trait and state emotion regulation and their interactions. In fact, given the strict relationship between expressive suppression, depression and stress-related symptoms (Moore et al., 2008), the question of whether this strategy is a vulnerability or causal factor remains still open. Otherwise, to evaluate its long term effects on anxiety, depression or other pathologies innovative clinical interventions could be designed training clients to cognitive reappraisal or even *positive reappraisal*, a recent trying to incorporate meditation mindfulness into cognitive therapy (Garland et al., 2009; Hanley and Garland, 2014).

Finally, another direction for the future studies is to carry out longitudinal researches that, allowing repeated observations of the effects of using particular emotion regulation strategies, would help to understand the causal order of effects of the habitual use of cognitive reappraisal or expressive suppression.

## Conflict of interest statement

The author declares that the research was conducted in the absence of any commercial or financial relationships that could be construed as a potential conflict of interest.
